# The Galectin Family as Molecular Targets: Hopes for Defeating Pancreatic Cancer

**DOI:** 10.3390/cells9030689

**Published:** 2020-03-11

**Authors:** Noemí Manero-Rupérez, Neus Martínez-Bosch, Luis E. Barranco, Laura Visa, Pilar Navarro

**Affiliations:** 1 Departament de Ciències Experimentals i de la Salut, Universitat Pompeu Fabra (UPF), 08003 Barcelona, Spain; noemi.manero@upf.edu; 2 Cancer Research Program, Hospital del Mar Medical Research Institute (IMIM), 08003 Barcelona, Spain; 3 Cancer Research Program, Hospital del Mar Medical Research Institute (IMIM), Unidad Asociada IIBB-CSIC, 08003 Barcelona, Spain; nmartinez@imim.es; 4 Department of Gastroenterolgy, Hospital del Mar-IMIM, 08003 Barcelona, Spain; lbarrancopriego@parcdesalutmar.cat; 5 Department of Medical Oncology, Hospital del Mar-IMIM-CIBERONC, 08003 Barcelona, Spain; lvisa@parcdesalutmar.cat; 6 Institute of Biomedical Research of Barcelona (IIBB-CSIC), 08036 Barcelona, Spain; 7 Institut d’Investigacions Biomédiques August Pi i Sunyer (IDIBAPS), 08036 Barcelona, Spain

**Keywords:** galectins, pancreatic cancer, stroma, immunosuppression

## Abstract

Galectins are a family of proteins that bind β-galactose residues through a highly conserved carbohydrate recognition domain. They regulate several important biological functions, including cell proliferation, adhesion, migration, and invasion, and play critical roles during embryonic development and cell differentiation. In adults, different galectin members are expressed depending on the tissue type and can be altered during pathological processes. Numerous reports have shown the involvement of galectins in diseases, mostly inflammation and cancer. Here, we review the state-of-the-art of the role that different galectin family members play in pancreatic cancer. This tumor is predicted to become the second leading cause of cancer-related deaths in the next decade as there is still no effective treatment nor accurate diagnosis for it. We also discuss the possible translation of recent results about galectin expression and functions in pancreatic cancer into clinical interventions (i.e., diagnosis, prediction of prognosis and/or therapy) for this fatal disease.

## 1. Pancreatic Cancer

The most common type of pancreatic cancer is pancreatic ductal adenocarcinoma (PDA), which is an aggressive disease with a devastating prognosis [1]. It is currently the third leading cause of cancer-related death and is expected to become the second by 2030 [2]. New and effective therapeutic strategies for pancreatic tumor patients are urgently required: PDA is normally diagnosed at late stages, with poor prognosis following standard chemo/radiotherapy [3].

A major hallmark of PDA is an abundant tumor stroma, which accounts for up to 90% of tumor volume and has been proposed to be one the main reasons for the aggressiveness of PDA and therapy inefficacy against it [4,5,6]. Stroma (also called the tumor microenvironment) mainly comprises extracellular matrix (ECM), cancer-associated fibroblasts (CAFs), endothelial cells, and immune cells. The majority of CAFs in PDA are activated pancreatic stellate cells (PSCs) [7]. In healthy pancreas, PSCs are quiescent and are characterized by lipid droplets containing vitamin A. PSCs become activated during cancer transformation, at which point they lose their lipid droplets and produce excessive ECM proteins, including collagen, fibronectin, laminin, α-smooth muscle actin (α-SMA), growth factors (such as platelet-derived growth factor (PDGF) and vascular endothelial growth factor (VEGF)), and several cytokines [7]. PSCs are activated by paracrine stimuli secreted by tumor cells, including TGF-β and sonic Hedgehog (Hh) [7,8]. Notably, ECM and secreted factors from activated PSCs also lead to increased pancreatic tumor cell motility, proliferation, metastasis, and chemoresistance, highlighting the relevance of tumor-stroma crosstalk in PDA progression [8,9,10].

Immune cells are also a very important component of tumor stroma. After the recent success of immunotherapy in some tumors [11,12], the PDA immune landscape has attracted high interest in pancreatic cancer research. Most pancreatic tumors display an immunosuppressive microenvironment, comprising M2 macrophages, myeloid-derived suppressor cells, and regulatory T-lymphocytes (Tregs), leading to impaired T-cell activation and inefficient cytotoxic T-lymphocyte-induced tumor elimination [13,14]. Pancreatic cells also contribute to immune system evasion by expressing and secreting immunosuppressive factors, such as TGF-β, IL-10, IL-6, VEGF, and the Fas ligand, among others [13,15]. Together, these processes lead to failed immune surveillance, which could explain the low rate of response to immunotherapy [9,14,15].

## 2. The Galectin Family

Galectins are a family of proteins that bind to carbohydrates, and in particular to β-galactose residues. Binding is mediated by a carbohydrate recognition domain (CRD) that is composed of 130 amino acids and that is conserved in all galectin types [16]. Although 15 galectins have been described in mammals, only 11 are found in humans [16]. Galectins are usually classified into three groups based on their structure: (i) prototypical galectins (galectin-1 (Gal1), Gal2, Gal5, Gal7, Gal10, Gal11, Gal 13, Gal14, and Gal15), characterized by a single CRD, which can act as monomers or form homodimers; (ii) the chimeric galectin Gal3 (the only member of this class), with a single CRD and a large amino-terminal domain that facilitates the formation of oligomers; (iii) the tandem repeat galectins, with two CRDs that are linked through a small peptide domain; this group includes Gal4, Gal6, Gal8, Gal9, and Gal12 [16].

Gal1 is expressed in endothelium, thymus, nervous system, and placenta among others [17,18,19]; Gal2 is expressed in smooth muscle cells and macrophages [20] and can be secreted in the gastrointestinal tract [21]; Gal3 is expressed in heart, kidney, vascular tissue, and macrophages [22]; Gal4 is mainly expressed in epithelial cells of the intestinal tract [23]; Gal9 is expressed in lymph nodes, bone marrow, thymus, and spleen [24,25] and Gal12 and Gal9 are expressed in adipose tissue [26]. Galectins are also involved in distinct physiological processes, such as embryogenesis, tissue regeneration, differentiation, vascularization, and immune response during inflammation and tolerance [16,27]. Depending on their cellular localization (nuclear, cytosolic, or extracellular), galectins can be involved in different functions, including RNA splicing, apoptotic signaling, endocytosis, glycan-binding mediated cell–cell or cell–ECM adhesion, and immune regulation, among others [28].

At the intracellular level, galectins are present both in cytosolic and nuclear location, where they can regulate several cellular functions. For instance Gal1 and Gal3 can interact with Ras or Bcl-2 regulating cellular growth and apoptosis [29]. They can also be secreted to the extracellular compartment through a non-canonical secretory pathway that is still not well understood [30,31]. Extracellularly, galectins can bind glycan structures such as disaccharide N-acetyllactosamine (LacNAc) present in glycosylated membrane receptors and ECM proteins. Glycan binding affinities are different for each galectin explaining why they have distinct biological activities [32]. The variation on glycan recognition are mainly due to N-glycan branching, multiplicity of LacNAc residues or terminal modifications such as fucosylation or sialylation [32]. In fact, it has been reported that integrin sialylation inhibits cell adhesion to Gal3 and modifies cell invasive capacity, likely via Rho-GTPase [33]. Moreover, O-glycan sialylation confers chemotherapy resistance by reducing the binding of Gal3 to cell surface glycosylated receptors in gastric cancer cells [34].

Gal1 was the first protein described in the galectin family. It binds to α2,3-sialylated or non-sialylated complex N-glycans containing poly-(LacNAc) residues. Gal1 can bind to proteins of the ECM, such as laminin and fibronectin. It is also found in the cell membrane of a wide variety of cell types bound to several receptors, such as integrin α5β1 in epithelial cells, GM1 glycolipid in neuronal cells, and CD45, CD43, and CD7 in immune cells [17,35]. Gal1 contributes to several cellular physiological functions, such as cell growth, migration, adhesion, motility, and T-cell homeostasis [17]. Gal3 is a 31-KDa protein and the only member of the galectin chimeric subfamily. It recognizes poly-LacNAc residues present in different glycolipids and glycoproteins and shows high specificity for internal LacNac motifs [36]. This lectin is mainly involved in cell proliferation, apoptosis [37], and in the immune system modulation [35,38]. Gal4 belongs to the tandem-repeat subfamily, with two CRD domains in a single peptide chain. It is mainly regulating lipid raft stabilization, protein apical trafficking, intestinal wound healing, neuron axon growth and myelination [23]. Gal4 also is involved in inflammatory intestinal diseases and some cancers [23,39]. Gal9 is a tandem repeat member that participates in tolerogenic macrophage programming and adaptive immune suppression [40], cytokine production [41] and T-cell signal transduction and apoptosis [42,43]. Gal9 has several partners, including dectin-1, TIM-3, and CD40, which are mainly involved in immunomodulatory effects [32,44]. Importantly, Gal9 has been described as an immune checkpoint [45].

## 3. Galectins in Cancer

Gal1 and Gal3 expression have been described to be altered in some tumors contributing to tumor cell proliferation, differentiation, and metastases [17,27,37,46]. In addition, Gal4 has been associated with many cancers having contradictory roles according to each cellular type, although its role has not been fully elucidated [23]. For instance, Gal4 is decreased in tumors and acts as a tumor suppressor in colorectal [47], hepatocellular [48], and pancreatic carcinomas [49], whereas it functions as a tumor promoter in lung cancer [50]. Finally, Gal9 expression has been reported in several solid tumors, such as melanoma [51], breast [52], and hepatocellular carcinomas [53], linking it to better prognosis and/or anti-metastatic potential due to its ability to block adhesion to endothelium and ECM [54]. Interestingly, in lung cancer, low Gal9 levels in tumor cells, or high Gal9 levels in T-lymphocytes, are correlated with poor prognosis [55]. Other studies have reported the relevance of Gal9/Tim-3 interaction in tumor immune evasion [56].

Interestingly, emerging data have demonstrated crucial functions of galectins, from both tumor and stroma cells, in PDA progression, such as a key role in modulating tumor immune escape, as we will describe in the next sections.

## 4. Galectin-1 and Its Role in Pancreatic Cancer

Gal1 is absent from healthy pancreas tissue; however, it is expressed in pancreatitis, and its expression increases in pancreatic tumors [57,58,59,60]. Notably, Gal1 expression in human PDA is restricted to tumor stroma, and, in particular, to activated PSCs [58,59,60,61,62]. Similar patterns of Gal1 expression are observed in genetically engineered mouse PDA models, where Gal1 levels are increased in the stroma of pancreatic tumors [62,63]. These data suggest that Gal1 plays a role in pancreatic cancer biology. In fact, the intensity of Gal1 staining of pancreatic tissue positively correlates with tumor size, perineural invasion, tumor stage, and metastases [58].

**Figure 1:**
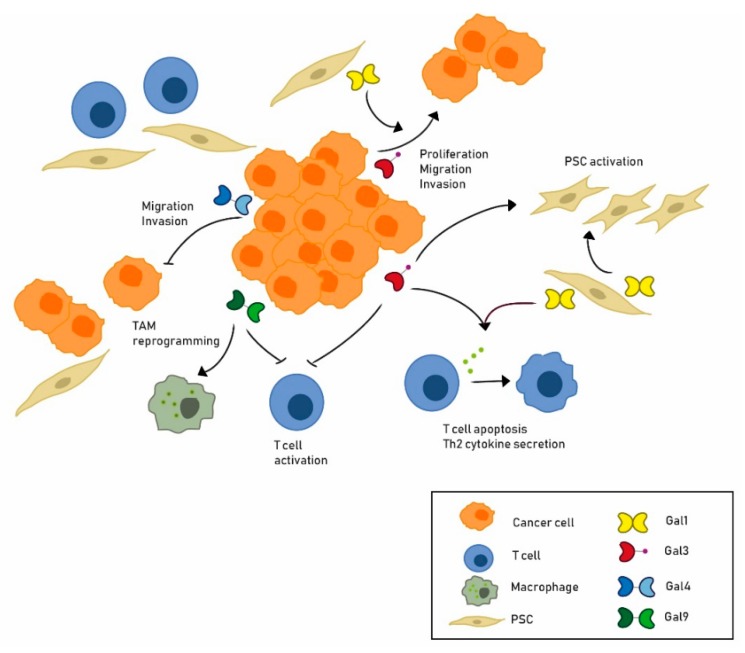
Expression and role of galectins in pancreatic cancer. The most important roles for Gal1, Gal3, Gal4, and Gal9 in pancreatic cancer are shown. Gal1, mainly expressed in pancreatic stellate cells (PSCs), has been reported to play an autocrine role in PSC activation as well as a paracrine role inducing tumor cell proliferation, invasion, migration, T-cell apoptosis, and Th2 cytokine secretion. Gal3, which is expressed in tumor epithelial cells, is associated to tumor cell migration, invasion, PSC activation, and immune response modulation. On the other hand, Gal4, expressed in tumor cells, inhibits tumor cell migration and invasion. Finally, Gal9 has been related to tumor-associated macrophage (TAM) reprogramming and inhibition of T-cell activation.

Gal1 expressed by activated PSCs has autocrine and paracrine functions in PDA (Figure 1, Table 1). Activated PSCs lose their fat droplets and increase their levels of α-SMA, an actin involved in fibrogenesis, and of Gal1, acquiring a more fibroblast like appearance [61,64]. In turn, downregulation of Gal1 in human activated PSCs reprograms these cells to a more quiescent phenotype [65]. Gal1-mediated activation of PSCs has been related to different signaling pathways, such as ERK, which is phosphorylated after Gal1 exposure [64,66], and TGFβ1/Smad [67]. Gal1 secreted by activated PSCs also induces paracrine effects in epithelial tumor cells. Increased invasiveness of the pancreatic cancer cell line CFPAC-1 is observed after exposure to recombinant Gal1 (rGal1) [61]. Similarly, migration and invasion of the RWP1 pancreatic cancer cell line was induced by conditioned media from human PSCs but not if they had been depleted for Gal1 [65]. Invasion and migration can be stimulated by increased levels of vimentin, MMP-9, and MMP-2 as well as by decreased levels of E-cadherin, induced by extracellular Gal1 in epithelial tumor cells [67,68]. Paracrine effects of Gal1 secreted by PSC are also observed in pancreatic tumor cell growth. Pancreatic tumor cells co-cultured with Gal1-overexpressing PSCs show significantly higher proliferation rates than those co-cultured in the presence of PSC with decreased Gal1 expression [61,67,68]. Likewise, conditioned media from human PSC increases RWP1 cell proliferation while Gal1 depleted PSC does not [65]. Further, rGal1 induces proliferation of pancreatic cancer cells [64,65].

Stromal-derived Gal1 also contributes in a paracrine way to modulating the immune microenvironment of pancreatic tumors (Figure 1, Table 1). Co-culturing of PSCs with T-lymphocytes induces CD3+, CD4+, and CD8+ apoptosis through caspase-9 and caspase-3, while Gal1 knockdown in PSCs correlates with increased CD4+ and CD8+ T-cell viability [57]. Moreover, Gal1 also alters T-cell cytokine secretion, favoring Th2 versus Th1 cytokines [57,69]. These in vitro data indicate that Gal1 may contribute to the pancreatic tumor immune escape. Indeed, Gal1 depletion in vivo re-establishes cancer immune surveillance, as explained below [62,65].

Using c-myc- or KRas-driven mouse models of PDA, and genetic depletion of Gal1 (Gal1 knockout (KO) mice), we have previously demonstrated that stromal Gal1 is a key mediator of pancreatic cancer development and progression (Table 1). Thus, Ela-myc tumors in a Gal1 KO background show a dramatic reduction of acinar-ductal metaplasia [62], a key event in the initiation of pancreatic malignant transformation [70]. Moreover, Gal1 depletion in these tumors strongly reduces tumor proliferation, angiogenesis, and stroma activation, while it increases necrosis and tumor-associated immune response [62]. Accordingly, Ela-myc mice KO for Gal1 have increased survival. Importantly, Gal1 induces Hh activation both in epithelial tumor cells and activated fibroblasts [62]. Along the same line, using a KRas-driven pancreatic cancer mouse model that faithfully recapitulates human PDA progression [71], our group has recently demonstrated that pancreatic tumors developed in a Gal1 KO background display longer latency and lower metastatic capacity, with an increase in animal survival of up to 20% [65]. These effects observed after Gal1 depletion are likely mediated by a multi-step mechanism involving decreased angiogenesis and stroma activation, as well as rescue of immune surveillance. In this regard, Gal1 KO pancreatic tumors have increased levels of CD3+, CD4+, and CD8+ T-effector cells together with decreased levels of CD11b+Gr1+ myeloid-derived suppressor cells, demonstrating that Gal1 overexpressed in PDA favors immune privilege [65].

The relevance of Gal1 in tumor-stroma crosstalk during PDA progression has also been demonstrated in xenograft experiments using human pancreatic tumor cells and activated PSC. Specifically, co-injection of tumor cells together with activated PSCs (which express high levels of Gal1) leads to increased tumor size and metastasis as compared to injecting tumor cells alone; correspondingly, depletion of Gal1 in the co-injected PSCs significantly reduces tumor growth and size [58,65,68]. Mechanistically, microarray studies have suggested candidate genes and pathways that are potentially involved in these effects on growth and metastasis, such as STAT and Hh signaling, IL1A, MMP-1, and ANK3 [62,65]. Moreover, increased metastasis formation could also be due to the axis SDF1-CXCR4 [72].

In summary, increasing evidence demonstrates that Gal1 is highly expressed in PDA, in particular by activated PSCs, and plays important roles in the tumor microenvironment crosstalk that drives pancreatic tumorigenesis and progression.

## 5. Galectin-3 and Its Role in Pancreatic Cancer

Gal3 is not detected in normal pancreas but is overexpressed in around 85% of PDA samples [73,74], correlating with human pancreatic disease progression [75]. Gal3 expression is also increased in chronic pancreatitis, although to a lesser extent than in PDA [75,76,77]. Further, in a KRas genetic mouse PDA model, Gal3 is overexpressed in pancreatic tumors but absent from normal tissue, mirroring the expression pattern found in humans [76]. Gal3 in PDA is mostly expressed in epithelial tumor cells, where it is mainly cytoplasmic with weakly detectable levels in nuclei and occasionally in stromal cells [73,74]. 

Similar to Gal1, Gal3 plays an important role in stimulating pancreatic cell proliferation both in an autocrine and in a paracrine way (Figure 1, Table 1). In vitro blockade of Gal3 using antibodies [78], interfering RNA [76,79], or chemical inhibitors [80,81] impairs proliferation, migration, invasion, and anchorage-independent growth of several pancreatic cancer cell lines, underscoring the contribution of Gal3 to tumor progression (Table 1). Secreted Gal3 also mediates tumor–stroma crosstalk impacting on PSC in a paracrine way (Figure 1). Thus, recombinant Gal3 (rGal3) added to PSC as well as Gal3 secreted by pancreatic tumor cells induces PSC proliferation [74,78]. In addition, conditioned medium from BxPC-3 overexpressing Gal3 increases PSC proliferation, migration, and invasion, whereas conditioned medium of PANC-1 downregulated for Gal3 has the opposite effect, as compared to conditioned medium from control cells [74]. Gal3 also induces PSC expression of α-SMA and ECM proteins, such as collagen1A1, collagen4A1, and fibronectin, indicating that Gal3 promotes PSC activation [74]. Moreover, PSC treated with rGal3 secrete more pro-inflammatory cytokines, such as IL8, IL6, CXCL1, CCL2, and GM-CSF, thereby contributing to maintaining their activated state and to promoting PDA progression (Figure 1). Interestingly, overexpression of Gal3 and some of these cytokines can also be observed in the human GEO database in patient samples as compared to control tissue [74].

In vivo, Gal3 inhibition has a significant impact on tumor growth (Table 1). Mice orthotopically injected with pancreatic cancer cells knocked down for Gal3 show smaller tumors than those injected with control cells. Immunohistochemical analysis of Gal3 knocked-down tumors confirmed that expression of Gal3 as well as of Ras and p-ERK, are reduced. Furthermore, metastases are only present in mice injected with control cells [76]. Along the same line, tumor growth and weight in mice were significantly reduced in PDAC xenografts as well as in PDAC patient-derived xenograft mice models after treatment with RN1—a polysaccharide Gal3 inhibitor—as compared to vehicle-treated mice [80]. Similar results were obtained with HH1-1, a novel Gal3 inhibitor: treating either mice injected with BxPC-3 or patient-derived xenograft mice with HH1-1 leads to reduced tumor sizes and weights [81]. Finally, Zhao et al. assessed another Gal3 inhibitor, MCP, that also impairs tumor growth and metastasis of PANC-1 injected mice [74].

These studies have shed some light on the mechanisms underlying Gal3-mediated functions in pancreatic cancer. In particular, after Gal3 silencing in PANC-1 cells, there is a reduction of Akt and GSK-3β phosphorylation and a subsequent downregulation of β-catenin expression, which results in decreased cell migration and invasion [79]. In addition, Gal3 binds and activates EGFR in pancreatic tumor cells, triggering MEK/ERK, BMP/Smad/Id-3, and integrin/FAK/JNK downstream signaling pathways [80]. Accordingly, the above-mentioned RN1 inhibitor blocks Gal3 binding to EGFR, thereby abolishing EGFR activation, decreasing ERK and MEK phosphorylation, and downregulating Runx1 levels (which in turn is a transcription factor of Gal3). Interestingly, MEK and Runx1 inhibition impairs pancreatic tumor cell growth, suggesting that ERK/Runx1 signaling is involved in the anti-tumor effect of this Gal3 inhibitor. Moreover, RN1 abolishes Gal3 binding to bone morphogenetic protein receptors (BMPR)-1A and -2 (thereby reducing Smad 1/5/8 phosphorylation and Id-3) and impairs Gal3 binding to integrins and subsequent FAK-JNK signaling [80]. In addition, and in line with data mentioned above, HH1-1 also impairs Gal3 binding to EGFR and subsequently blocks the EGFR/AKT/FOXO3 pathway and decreases levels of Smad1/5/8, Id-1, and β-catenin [81]. Altogether, these results indicate that the pro-tumor effects of Gal3 in pancreatic cancer epithelial cells are mediated through a crosstalk of these different signaling pathways. Moreover, Gal3-mediated induction of IL8 secretion in PSCs is suppressed by an NF-kB inhibitor, indicating that Gal3 stimulates IL8 secretion by PSCs through the NF-kB signaling pathway [74]. Another study has reported that Gal3 can interact with LI-cadherin, a unique member of the cadherin superfamily that is detected in 82% of pancreatic carcinomas and positively correlates with tumor differentiation and increased survival. Remarkably, these two proteins co-localize by immunohistochemistry in PDA tissue sections, suggesting that Gal3–LI-cadherin binding plays a role in pancreatic cancer [82].

Gal3 has a key role in tumor immune escape by affecting different immune cell types [32,83] and has been introduced to the list of immune checkpoints due to its interaction with LAG-3 and links to T-cell exhaustion [45]. Importantly, Gal3 is involved in TCR clustering [84] and induces T-cell apoptosis via interactions with CD45 and CD71 [85]. Further, it is a marker of M2 macrophage activation [86,87] and maintains a macrophage fate by binding to CD98 and triggering PI3K activation [86]. Interestingly, in preclinical trials, Gal3 blockade in solid tumors together with PD1/PDL1 checkpoint inhibition or T-cell agonists boost an immunologic response against tumors, resulting in tumor regression [88].

Gal3 impairs IFN-Ƴ secretion by CD4+ and CD8+ T lymphocytes; indeed, GCS-100 combined with therapeutic vaccination promotes cytotoxicity and cytokine secretion, resulting in increased tumor rejection [88]. Interestingly, ascites of pancreatic cancer patients were included in the work of Demotte et al. proving in vitro that GCS-100 boosts IFN-γ secretion by CD8+ TILS, which exhibited significant cytotoxicity [88] (Table 1). Gal3 also popped up as a target of the immune response when Kouo and colleagues interrogated sera from PDA patients enrolled in a phase II study before and after receiving GM-CSF vaccines and compared responders to non-responders [38] (Table 1). Intriguingly, purified IgGs only from post-vaccine patient serum attenuated IFN-Ƴ reduction induced by rGal3, due to the presence of neutralizing antibodies against this protein [38].

Therefore, Gal3 is overexpressed in PDA and mainly secreted by tumor cells, promoting tumor progression by autocrine direct effects on tumor cell proliferation, invasion, and migration as well as paracrine effects in the stroma by promoting activation of PSCs and negative modulation of anti-tumor immune response.

## 6. Galectin-4 and Its Role in Pancreatic Cancer

In pancreas, Gal4 levels are almost absent from normal tissue, whereas its strong expression has been reported in pancreatic cancer cell lines and tumors. Gal4 staining is found in 80% of tumor cells, mainly localized in cytoplasmic and nuclear compartments. Kuhlmann et al. reported that Gal4 is overexpressed in the exocrine molecular subtype of pancreatic ductal adenocarcinoma [89]. Interestingly, Gal4 levels in human tumors inversely correlate with the presence of lymph node metastases: 70% of patients with lymph node metastases have low levels of Gal4, while those with high Gal4 levels display less metastases. Moreover, tumors with lower Gal4 levels show a tendency to have increased vascular infiltration [49]. Importantly, Gal4 expression positively correlates with 1- and 3-year overall survival [90]. These data suggest that, in contrast to Gal1 and Gal3, Gal4 may play a tumor suppressor role in PDA.

Studies using human PDA cell lines also support a tumor suppressor role for Gal4 (Figure 1, Table 1). Pa-Tu-8988S, a pancreatic cell line with high Gal4 levels, has a low metastatic capacity as compared to Pa-Tu-8988T cells, which has low Gal4 levels [91]. Overexpressing Gal4 in Pa-Tu-8988T significantly decreases in vitro migration in scratch assays as compared to mock cells. In vivo, in zebrafish embryos, xeno-injection of Pa-Tu-8988T cells overexpressing Gal4 leads to a significant reduction of metastasis as compared to injecting mock cells [91]. Likewise, an increase of metastases is observed in zebrafish embryos transplanted with Pa-Tu-8988S siRNA Gal4 cells as compared to mock cells [91]. These data have been extended using primary cells from PDA patients with different levels of Gal4. PDAC-1 cells expressing high levels of Gal4 are less invasive and less migratory than PDAC-2 cells, which express lower levels of Gal4 [49]. Correspondingly, Gal4 overexpression in PDAC-2 cells reduces their migration and invasion capacity compared to control cells while Gal4 downregulation by siRNA in PDAC-1 increases these activities (Figure 1). Accordingly, in vivo mouse pancreatic orthotopic injection results in more macroscopic metastases and lower survival times when PDAC-2 cells are injected versus PDAC-1 cells. Interestingly, Gal4 expression correlates with reduced levels of β-catenin and Wnt signaling inhibition, suggesting that the impairment of migration and invasion induced by Gal4 are mediated through negative regulation of the Wnt/β-catenin pathway [49].

In conclusion, Gal4 overexpression in PDA correlates with better prognosis and survival as well as with a reduction of tumor cell migration, invasion, and metastasis, therefore suggesting its role as a tumor suppressor in pancreatic cancer.

## 7. Other Galectins and Their Role in Pancreatic Cancer

The expression and roles of other galectin members in PDA have been poorly explored. Danguy et al. found lower levels of Gal8 in malignant pancreatic tumors compared to normal or benign pancreatic tissue, suggesting Gal8 as a possible immunohistochemical diagnostic marker for pancreas malignancy [92]. On the other hand, Gal7 levels are upregulated in gemcitabine-treated pancreatic tumor cells as compared to non-treated cells, suggesting a possible role for Gal7 in chemotherapy resistance [93].

Gal9 has also been studied in PDA progression, as it is expressed in both leukocytes and tumor cells [40]. Anti-Gal9 antibodies significantly produce tumor regression of PDA cells injected subcutaneously into mice and increases survival in a KPC mice model that expresses Gal9 in cancer cells and some intratumoral myeloid cells [40]. Interestingly, either Gal9 or PD1 blockade results in a better T-cell activation. In fact, Gal9 blockade enhances intra-tumoral T-cell activation in PDA but not when dectin-1 (a Gal9-binding partner present in macrophages) is deleted, indicating that a Gal9/dectin axis is involved in CD4+ and CD8+ T-cell reprogramming [40]. Gal9 inhibition is also associated with immunogenic reprogramming of tumor-associated macrophages (TAMs) in PDA (Figure 1, Table 1) [40]. Finally, Gal9 is involved in PDA immunotherapy resistance, with recent data showing that Gal9 inhibition enhances chimeric antigen receptor (CAR) T-cell cytotoxicity [94] (Table 1).

## 8. Clinical Opportunities

Taking into account the relevance of galectins in PDA progression, their frequent overexpression in this tumor, and their hallmark of being soluble secreted molecules, these proteins emerge as promising biomarkers for pancreatic cancer as well as novel therapeutic targets.

Gal1 is often upregulated in PDA tissues as compared to normal tissues, and it correlates with histology, T-stage, and N-stage [95]. Low expression of stromal Gal1 detected by immunostaining in PDA tissue samples is associated to long-term survival [96], highlighting the potential of Gal1 expression as a biomarker for pancreatic cancer diagnosis and prognosis. Interestingly, plasma Gal1 levels detected by ELISA are found to be significantly increased in patients with PDA as compared to a control group, underscoring the potential of using circulating Gal1 levels as a diagnosis biomarker [59]. In addition, using a combination of Gal1 and CA19-9 markers decreases the ratio of false-negatives and increases sensitivity and specificity, suggesting that Gal1 could be used as a complementary plasma biomarker for PDA diagnosis [59]. High Gal1 levels are related to lower patient survival [59,96,97], suggesting a role for circulating Gal1 as a prognostic marker. Similarly, Gal3 is overexpressed in PDA tissue samples and not expressed in normal pancreas [73,74]. Gal3 levels in plasma of PDA patients have been found to be significantly higher than in a control group, suggesting also the possible role of Gal3 as a biomarker [73]. Finally, serum samples from PDA patients have higher levels of Gal9 than healthy controls, suggesting also new diagnosis roles for Gal9 [98].

In addition to potential roles as cancer biomarkers, galectins also present a huge potential for developing novel therapies for PDA. In fact, the in vitro and in vivo results discussed above that have used genetic strategies to inhibit different galectin members (and in particular, Gal1, Gal3, or Gal9) have consistently demonstrated that depletion of these proteins impairs tumor progression, building the foundations for developing pharmacological inhibitors that could translate these results to cancer patients. Galectin inhibitors have recently emerged as exciting new therapeutic approaches for the treatment of different tumors, either as single agents or in combination with other therapies. These new drugs may involve specific monoclonal antibodies, small molecules, or competitive carbohydrate derivatives. OTX008, a selective small molecule Gal1 inhibitor, has been preclinically tested and shown to inhibit tumor growth in human glioblastoma and ovarian cancer [99], head and neck squamous carcinoma [100], and hepatocellular carcinoma [101]. These results have led to a phase I clinical trial to evaluate the effects of OTX008 in advanced solid tumors [102]. Another galectin inhibitor, GM-CT-01 (DAVANAT), a modified galactomannan oligomer that binds Gal1 and Gal3, in combination with 5-FU, successfully completed phase II clinical trials for patients with colon cancer showing increased survival and reduced serious adverse effects compare to Best Standard of Care [103]. In addition, GR-MD-02 (a modified version of GM-CT-01) is currently being testing in combination with anti-PD1/PDL1 immune checkpoints in melanoma patients with encouraging preliminary results [104,105] reported by the company. Some inhibitors of Gal3, such as G3-C12, which have been tested in breast, colon, and prostate xenografts have been shown to reduce metastasis and lead to tumor regression [102]. GCS-100, which is derived from modified citrus pectin, also inhibits Gal3 and has been tested in a phase II clinical trial of patients with chronic lymphocytic leukemia, with an overall good tolerance and partial response in some patients [102]. Despite these encouraging results, further studies and the inclusion of patients with PDA are required to better understand the impact of galectin inhibitors in the treatment of this disease.

Taking into account the galectin panel and their altered expression in pancreatic cancer, together with the hints derived from preclinical studies, there is rationale to propose galectins as plausible targets in this tumor type. Besides, several studies have suggested a possible synergistic effect of targeting galectins together with chemotherapy administration in cancer. Interestingly, it has been reported that chemotherapy can induce Gal3 which participates in multidrug resistance processes by binding to Na+/K+-ATPase and P-glycoprotein [106,107]. Moreover, Gal1 downregulation in lung and ovarian cancer cells by shGal1 and siGal1, respectively, have shown to significantly decrease viability after cisplatin treatment in comparison to control transfected cells, suggesting that Gal1 depletion enhances sensitivity to cisplatin [108,109]. Similarly, Gal3 downregulation by siRNA enhances chemotherapy response of pancreatic cell lines in vitro. Moreover, in vivo gemcitabine treatment of pancreatic tumor xenografts shows higher reduction of tumor burden after Gal3 siRNA intratumoral injection [110]. These data suggest that the combination of Gal1 or Gal3 inhibitors with standard chemotherapy could enhance its efficacy and benefit PDA patients.

Galectins have also been related to immunotherapy resistance. Intriguingly, due to the contribution of some galectins in conferring immune privilege, targeting these lectins or the galectin/ligand axis in tumors, either alone or in combination with other therapies, could potentially restore the anti-tumor immunity. Indeed, Gal1 selectively recognizes and eliminates Th1 and Th17 cells due to their particular glycosylation pattern [111]. Remarkably, blockade of Gal1 with monoclonal antibodies increase CD4+ CD8+ tumor infiltrating lymphocytes in melanoma [112] and lung cancer through T-cell proliferation and the induction of tumor-specific T1-type immunologic response in lymph nodes [35]. Gal1 is also known to mediate CD20 immunotherapy resistance in a preclinical mouse model of non-Hodgkin lymphoma. The high levels of this lectin in human lymphomas suggest that Gal1 may also hamper CD20 immunotherapy in patients [113]. Recent data have shown that head and neck cancer can have Gal1-driven resistance to immunotherapy, and that Gal1 inhibition with CRISPR/Cas9 or an antibody enhances anti-PD1 therapy, suggesting that a combination of Gal1 inhibitors and α-PD1/PDL1 immune checkpoint synergize for cancer treatment [114]. Resistance to immunotherapy mediated by Gal1 takes place through upregulation of PD-L1 and Gal9 in the endothelium, resulting in impaired T-cell infiltration [114]. In lung cancer, accumulation of Tim-3-expressing lymphoid cells and Gal9-expressing monocytic myeloid derived suppressor cells positively correlates with resistance to anti-PD1 immunotherapy. Accordingly, resistance to anti-PD-1 can be overcome by in vitro blockade of the Gal9/Tim3 pathway [115]. Emerging data are also addressing how Gal3 inhibition could potentiate immunotherapy efficiency. For instance, treatment with a small molecule inhibitor targeting Gal3 (GB1107) boosts anti-PD-L1 therapy in lung adenocarcinoma preclinical assays by promoting CD8+ T-cell infiltration and M1 macrophage polarization [116]. In melanomas, ex vivo expansion of T-cells for adoptive cell transfer therapy was directly related to the amount of Gal3 secreted by tumor cells and blocking the lectin enhanced T-cell activation [117].

Finally, several phase I clinical trials have been designed to treat patients with Gal3 inhibitors together with immunotherapy in different tumor types, including, non-small cell lung cancer, squamous cell head and neck cancers [105], and melanoma [104], the latter of which has given promising results.

Altogether, PDA preclinical studies together with ongoing clinical trials in other tumors indicate that galectin inhibitors can offer promising opportunities for pancreatic cancer therapeutic interventions, either alone or combined with current chemo- and/or immunotherapies. While this is potentially great news, it is still too soon to celebrate, as important aspects first need to be taken into consideration. For example, development of selective inhibitors is challenging because several galectin members display high homology, in particular in their CRDs, which play a major role in ligand recognition. Moreover, tumors can express different galectin members with overlapping functions; therefore, tumors can compensate the blockade effects of selective inhibitors by upregulating another galectin with similar functions. In fact, this has been reported for Gal1 and Gal3 in PDA [118]. Therefore, we first require an exhaustive information base covering the entire picture of galectins and their specific functions in PDA. On the other hand, galectin inhibition using broad spectrum inhibitors (i.e., inhibiting more than one member) may result in severe adverse effects especially given the key physiological role of these proteins in immune system regulation. Thus, although translation of preclinical studies in PDA using galectin blockade seems promising for patients, we should proceed with caution when considering if and how to implement this strategy for clinical interventions. In contrast, analyzing galectins (from either tissue samples or blood) as novel methods for diagnostics, prognostics, or even predictors of therapy response biomarkers seems to be a more realistic strategy for rapidly translating the already generated basic research to bedside.

## Figures and Tables

**Table 1:** The expression of galectins and their main functions in pancreatic ductal adenocarcinoma (PDA).

Galectin	Role	Expression	Functions	References
Gal1	Pro-tumoral	Stroma	Increase cell proliferation	[58,61,62,64,65,66,67,68]
			Induce migration and invasion	[58,61,65,66,67,68]
			Enhance angiogenesis	[62,65]
			Immune evasion	[57,62,65,69]
			Induce acinar to ductal metaplasia	[62]
Gal3	Pro-tumoral	Tumor epithelial cells	Increase tumor cell growth	[74,76,78,79,80,81]
			Increase migration and invasion	[74,76,78,79,80,81]
			Immune escape	[38,88]
Gal4	Tumor suppressor	Tumor epithelial cells	Decrease migration and invasion	[49,90,91]
Gal9	Pro-tumoral	Tumor epithelial cells	Immune reprogramming	[40,94]
